# *In vitro* antioxidant and, α-glucosidase inhibitory activities and comprehensive metabolite profiling of methanol extract and its fractions from *Clinacanthus nutans*

**DOI:** 10.1186/s12906-017-1684-5

**Published:** 2017-03-31

**Authors:** Md. Ariful Alam, I.S.M. Zaidul, Kashif Ghafoor, F. Sahena, M. A. Hakim, M.Y. Rafii, H.M. Abir, M.F. Bostanudin, V Perumal, A. Khatib

**Affiliations:** 1 Faculty of Pharmacy, International Islamic University Malaysia (IIUM), Kuantan Campus, 25200 Kuantan, Pahang Malaysia; 2 Department of Food Science and Nutrition, King Saud University, Riyadh, 11451 Saudi Arabia; 3 Faculty of Science, International Islamic University Malaysia (IIUM), Kuantan Campus, 25200 Kuantan, Pahang Malaysia; 4 Institute of Tropical Agriculture, University Putra Malaysia, 43400 UPM, Serdang, Selangor Darul Ehsan Malaysia; 5 Faculty of Agricultural Engineering and Technology, Bangladesh Agricultural University, Mymensingh, Bangladesh; 6 Department of Pharmaceutical Technology and Industry, Faculty of Pharmacy, Cyberjaya University College of Medical Science, No. 3410, Jalan Teknokrat 3, Cyber 4, 63000 Cyberjaya, Selangor Darul Ehsan Malaysia

**Keywords:** Clinacanthus nutans, Antioxidant activity, α-glucosidase inhibitory activity, Reducing power, Total phenolic, Total flavonoid, Activity phytochemical profiling

## Abstract

**Background:**

This study was aimed to evaluate antioxidant and α-glucosidase inhibitory activity, with a subsequent analysis of total phenolic and total flavonoid content of methanol extract and its derived fractions from *Clinacanthus nutans* accompanied by comprehensive phytochemical profiling.

**Methods:**

Liquid-liquid partition chromatography was used to separate methanolic extract to get hexane, ethyl acetate, butanol and residual aqueous fractions. The total antioxidant activity was determined by 2,2-diphenyl-1-picrylhydrazy (DPPH) radical scavenging and ferric reducing antioxidant power assay (FRAP). The antidiabetic activity of methanol extract and its consequent fractions were examined by α-glucosidase inhibitory bioassay. The chemical profiling was carried out by gas chromatography coupled with quadrupole time-of-flight mass spectrometry (GC Q-TOF MS).

**Results:**

The total yield for methanol extraction was (12.63 ± 0.98) % (*w*/w) and highest fractionated value found for residual aqueous (52.25 ± 1.01) % (*w*/w) as compared to the other fractions. Significant DPPH free radical scavenging activity was found for methanolic extract (63.07 ± 0.11) % and (79.98 ± 0.31) % for ethyl acetate fraction among all the fractions evaluated. Methanol extract was the most prominent in case of FRAP (141.89 ± 0.87 μg AAE/g) whereas most effective reducing power observed in ethyl acetate fraction (133.6 ± 0.2987 μg AAE/g). The results also indicated a substantial α-glucosidase inhibitory activity for butanol fraction (72.16 ± 1.0) % and ethyl acetate fraction (70.76 ± 0.49) %. The statistical analysis revealed that total phenolic and total flavonoid content of the samples had the significant (*p* < 0.05) impact on DPPH free radical scavenging and α-glucosidase inhibitory activity.

**Conclusion:**

Current results proposed the therapeutic potential of *Clinacanthus nutans*, especially ethyl acetate and butanol fraction as chemotherapeutic agent against oxidative related cellular damages and control the postprandial hyperglycemia. The phytochemical investigation showed the existence of active constituents in *Clinacanthus nutans* extract and fractions.

## Background

The chemotherapeutic uses of medicinal plants are a result of ancient people’s belief in their protective effects. Many plants have abundant active secondary metabolites that exhibit certain pharmacological effects in humans. Thus plant based traditional remedies have been developed from these chemotherapeutic agents to prevent certain ailments [1]. These active constituents are varied in their chemical structure, chemical composition, protective activity, and mode of action, hence, medicinal herbs have been used in traditional medicine. Antioxidant-rich natural products in the form of herbal formulations are able to interrupt cellular damage, mainly through the mechanism of free radical scavenging [2, 3]. Free radical causes oxidative stress related-complications in the human body owing to a physiological imbalance of free radicals and antioxidants, which is related to the impairment of primary metabolites, and consequently result in a number of health impediments [4, 5]. Phenolic and flavonoid compounds are the most common antioxidant metabolites in plants and include saponins, alkaloids, coumarins, tannins, lignin, terpenoids, stilbenes, quinones, amines and betalains [6, 7]. These compounds are important active natural products, which possess various biological actions, such as anti-diabetic, anti-inflammatory, hepatoprotective, anti-allergic, anti-carcinogenic, anti-thrombotic, anti-microbial activities, anti-atherosclerotic, anti-tumor and anti-mutagenic activity [8, 9].

Diabetes is a pandemic endocrine disorder characterized by hyperglycemia resulting from insulin resistance or overall insulin dysfunction [10, 11]. Modern synthetic hypoglycemic agents can cause side effects, are costly and decline in efficiency with long term uses [12, 13]. The inhibition of the α-glucosidase enzyme can control the intestinal carbohydrate metabolism by retarding the cleavage of polysaccharides to glucose [14, 15]. Therefore, the best strategy is to reduce the postprandial upsurge of blood glucose level and prevent late diabetic complications. Thus natural product based α-glucosidase inhibitors are the key targets to identify new compounds for the therapeutic management of diabetes [16]. The extensive literature reported more than four hundred natural products isolated from various herbal plants that exhibited digestive enzyme inhibitory activity [17], a majority of the compounds were flavonoid, terpene, and phenylpropanoid ring containing compounds [17].

*Clinacanthus nutans* Lindau is a medicinal plant from Acantheceae family widely distributed throughout tropical Asia. For a long time, this shrub has been used to prevent skin infections, insect bites and lesions caused by the herpes simplex and varicella-zoster viruses [18, 19]. Many beneficial effects of this plant have been reported, including antidiabetic, antioxidant, anti-proliferative, immunomodulatory, wound healing, anti-inflammatory, analgesic activities and anti-viral activities [20–23]. However, its efficacy is yet to be proven scientifically and more research needs to be carried out, especially with regard to the antidiabetic effects of this plant. Our analysis of the current literature ascertained that- the free radical scavenging activity, ferric reducing antioxidant power, and α-glucosidase inhibitory activity of the methanol extract and its different polar, non-polar fractions from *C. nutans* have not been previously determined. Moreover, the complete phytochemical profile of this plant remains unestablished. Therefore, this study aimed to investigate the *in vitro* antioxidant and α-glucosidase inhibitory activities of methanol extract and its different fractions from *C. nutans*. Our research also identified the various chemical constituents of the extract and fractions by GC Q-TOF MS, in addition to bioactivity correlation.

## Methods

### Sample collection and preparation

The stem of *C. nutans* was obtained from TKC Herbal Nursery SDN BHD, Pusat Pertanian Pantai Baru, Pantai Negeri Sembilan, Malaysia in September 2014. The plant material was identified by Dr. Alfi Khatib, Associate Professor, Department of Pharmaceutical Chemistry, International Islamic University, Kuantan, Pahang, Malaysia. A voucher specimen (PIIUM 0238–1) was deposited in the herbarium of the Faculty of Pharmacy, International Islamic University, Kuantan, Pahang, Malaysia. The *C. nutans* stem was dried, in an airy place under shade. When dry the samples were ground in a cutting mill (FRITSCH, Pulverisette 19, Germany), sieved, and fractionated by a vibratory sieve shaker (AS 700, BASIC, RETSCH, Germany) to obtain fine particles. The extracts and fractions were freeze dried before analysis of the pharmacological activity and chemical profile (Alpha 1–2 LD plus CHRIST, Freeze dryer, UK).

### Chemicals

Ethanol (99.5%, analytical grade), Folin-Ciocalteu phenol reagent, potassium acetate, aluminum chloride, sodium carbonate, methanol, hexane, ethyl acetate and butanol were obtained from Merck Germany (Darmstadt, Germany). α-Glucosidase enzyme (*Saccharomyces cerevisiae*) and 4-nitrophenyl β-D-glucopyranoside substrate were obtained from the Sigma Chemical Co (St. Louis, Mo., U. S. A.). 2, 2-Diphenyl-1-picrylhydrazyl (DPPH), methoxyamine hydrochloride, pyridine, *N*-Methyl-N-(trimethylsilyl) trifluoroacetamide, quercetin (≥95% HPLC, solid), and rutin hydrate (≥94% HPLC, powder) were obtained from Malaysia Sigma-Aldrich (M), Sdn. Bhd. Kuala Lumpur, Malaysia. All other chemicals used were of analytical grade.

### Extraction and fractionation

The solvent extraction of the air-dried stem of *C. nutans* was performed by maceration technique using methanol as the initial solvent in an Erlenmeyer flask. After maceration, the mixture was sonicated for 15 min, and set aside for 24 h, filtered, and evaporated in a rotary evaporator at 40 °C under reduced pressure. The extract was freeze dried and stored ready for pharmacological analysis and fractionation.

The fractionation of dried methanolic extract was achieved by liquid-liquid partition chromatography in a separating funnel using hexane, methanol, and water (13:2:5) with a final volume of 2 L, based on the amount of extract. After vigorous shaking, the mixture was set aside until two layers were formed. To obtain the hexane fraction, the was separated and then concentrated in rotary evaporator at 40 °C under reduced pressure. To obtain the ethyl acetate fraction, the remaining fraction in the separating funnel was added to ethyl acetate and the evaporation procedure was repeated. Similarly, butanol was added to obtain the butanol fraction; the remaining material in the separating funnel was considered as the residual aqueous fraction. The residual solvent was removed from the extract and fractions by freeze drying. The methanol extract and its four fractions were then freeze-dried and stored at −80 °C freezer until use.

### Total phenolic content

The total phenolic content (TPC) of the methanol extract and the derived fractions from *C. nutans* were determined spectrophotometrically according to the Folin-Ciocalteu method [24]. The reaction mixture was prepared by mixing 20 μL of the extract solution (at 5 mg/mL in DMSO), 100 μL Folin-Ciocalteu reagent (1 mL of Folin-Ciocalteu reagent in 9 mL of distilled water) and 80 μL 7.5% Na_2_CO_3_ solution in deionized water. The solution was incubated for 30 min in a dark place at 26.8 °C and the absorbance was measured at 765 nm. The total phenolic concentration was calculated from a gallic acid (GA) calibration curve 10–100 mg/L; *y* = 0.0005*x* + 0.0779, R^2^ = 0.9943. Data were expressed as gallic acid equivalent/g of extract averaged from three measurements.

### Total flavonoid content

The total flavonoid content (TFC) of the *C. nutans* extract and fractions were estimated according to the aluminum chloride spectrophotometric method based on the formation of aluminum-flavonoid complexes [25]. To prepare the reaction solutions, approximately 2 mL extract solution (0.3 mg in 1 mL of methanol), 0.1 mL aluminum chloride hexahydrate solution (10% aqueous AlCl_3_ solution), 0.1 mL 1 M potassium acetate and 2.8 mL of deionized water were mixed together. The mixture was shaken and incubated at 26.8 °C for 10 min. After incubation, the solution was subjected to spectral analysis at 415 nm. A standard curve was constructed of the absorbance of rutin between 0.005 and 0.1 mg/mL and the total flavonoid content calculated as mg rutin equivalent per g dry extract. The absorbance at 415 *nm* = 14.171*x* + 0.0461, R^2^ = 0.9991.

### DPPH free radical scavenging activity

The free radical scavenging capacity of the methanol extract and each derived fractions from *C. nutans* were analyzed by using a 96-well microplate and 2, 2-diphenyl-1-picrylhydrazyl (DPPH) as the free radical source [26]. For sample preparation, 5 mg freeze dried sample was added to 1 mL of DMSO, which was vortexed and sonicated to produce a homogenized solution. The sample solution was transferred (100 μL) to a 96well plate and 50 μL of this sample was diluted with 50 μL DMSO. Then, 100 μL DPPH (5.9 mg in 100 mL 100% ethanol) was added to each of dilutions, suspended and incubated in a dark place for 30 min. Blank samples were prepared following the same procedure, but used using 100 μL 100% ethanol instead of DPPH. A reagent blank which contained 50 μL DMSO and 100 μL DPPH, but no sample was, also subjected to the sample preparation process. Quercetin was used as a positive control to observe the reaction of inhibition. The absorbance was recorded at 517 nm and the percentage inhibition was calculated by using the following equation-

1 $$ \% Inhibition=\frac{R_b-\left({S}_a- B{S}_a\right)}{R_b}\times 100 $$

*R*_*b*_ = Reagent blank absorbance

*S*_*a*_ = Sample absorbance

*BS*_*a*_ = Blank sample absorbance

### Ferric reducing antioxidant power assay

The total antioxidant capacity of methanol extract and other derived fractions were examined by using the FRAP method adapted by Szd lowska Czerniak *et al.,* with some modifications [27]*.* FRAP reagent (10 mM TPTZ solution) (2.5 mL) in 40 mM HCl, 20 mM FeCl_3_ (2.5 mL), and 0.1 M acetate buffer pH 3.6 (25 mL) was freshly prepared and incubated for 10 min at 37 °C in an oven. Then 20 μL of different concentration (0%, 20%, 40%, 60%, 80% and 100%) of methanol extract and fractions of *C. nutans* and ascorbic acid (standard) and 40 μL FRAP reagent were added to 140 μL of distilled water in a 96 wells plate. The samples were stored at 26.8 °C for 20 min in dark and the blue color was quantified through measurement of the absorbance at 593 nm relative to a reagent blank (40 μL FRAP reagents and 160 μL distilled water) using microplate reader. Ascorbic acid was used as a standard compound to create a calibration curve. The total antioxidant capacity of the samples was expressed in ascorbic acid equivalents as (AAE) in μg/g of dried sample.

### α-Glucosidase inhibitory assay

The α-glucosidase inhibitory activity of the *C. nutans* extract and each of the fractions from liquid-liquid partition chromatography was determined following the method by Collins *et al.,* [28]. In this assay, the formation of *p*-nitrophenol that resulted from the cleavage of *p*-nitrophenyl-α-D-glucopyranose was estimated for the evaluation of α-glucosidase inhibitory activity. To prepare the stock solution, 5 mg of the extract and fractions were dissolved in 1 mL of DMSO. Additionally, 5 mg quercetin was dissolved in 1 mL of DMSO to form a positive control [29]. For the dilution, 10 μL prepared stock solutions were added to 115 μL 30 mM phosphate buffer (pH 6.5). The enzyme (α-glucosidase type 1 from *Saccharomyces cerevisiae*) (Sigma G5003) solution was prepared using 1 mg enzyme with 13.9 mL 50 mM phosphate buffer (pH 6.5). The substrate containing 3 mg *p*-nitrophenyl-α- D-glucopyranose (Sigma-Aldrich, N1377-1G) was dissolved in 10 mL of a 50 mM phosphate buffer (pH 6.5) and incubated at 26.8 °C for 15 min. The diluted samples were mixed with 15 μL of enzyme and 75 μL of substrate in the microplate, and incubated for 15 min at 26.8 °C. A blank sample, positive control, negative control, and blank positive control were prepared according to the mentioned method. Glycine solution was prepared as a reaction-stopping reagent by the dissolution of 15 g with 100 mL of cold water (pH 10). The optical densities (ODs) were read at 405 nm in a multi-detection micro-plate reader (Infinite M200, Tecan, Switzerland) [30]. The inhibitory activity was calculated according to the following equation

2 $$ \textcolor[rgb]{0.07450980392156862,0.0784313725490196,0.07450980392156862}{\kern0.75em }\% Inhibition\  of\  sample=\frac{a_n-{a}_s}{a_n}\times 100 $$

where *a*_*n*_ = negative control and *a*_*p*_ = (Sample Absorbance-Blank Sample Absorbance).

### Derivatization for GC Q-TOF MS analysis

Derivatization of the methanol extract and the derived polar-nonpolar fractions from *C. nutans* were performed by methoxyamination and trimethylsilylation (TMS) for GC Q-TOF MS chemical profiling according to the method mentioned by Robinson *et al.* [31]*.* The methoxyamine hydrochloride was dissolved in pyridine to produce a concentration of 20 mg/mL. Exactly 2.5 mg of sample was mixed with 50 μL pyridine in a 2-mL centrifuge tube and sonicated for 10 min at 30 °C. Then, 100 μL methoxyamine hydrochloride (20 mg/mL in pyridine) was added to the sample solution and incubated for 2 h at 60 °C. After the incubation 300 μL MSTFA (*N*-Methyl-*N*-(trimethylsilyl) trifluoroacetamide) was mixed and incubated for a further 30 min at 60 °C. The prepared sample was stored overnight at 23 °C for the completion of chemical reaction. The mixture was then filtered by syringe filter for the separation of any solid particles.

### GC Q-TOF MS determination

The GC-MS Agilent system of model 7200 accurate-mass GC QTOF equipped with a 7890A GC system connected to a detector quadrupole time of flight mass spectrometer was used to acquire mass spectral data. Exactly, 1 μL of derivatized sample was injected into the inlet of the Agilent GC column (J &W Scientific, Folsom, CA, USA) model, HP-5MS; dimensions 30 m × 0.25 mm × 0.25 μm), with 50:1 split mode and ratio. The injector temperature was maintained at 280 °C and the detector was maintained at 290 °C. The oven temperature profile was as follows: an increase from set to 70 to 135 °C with a 2 °C/min, hold for 10 min, an increase from 135 to 220 °C with 4 °C/min, hold for 10 min, an increase from 220 to 270 °C at 3.5 °C/min and then a final hold for 20 min. Helium was used as carrier gas at a constant flow rate of 1.9 mL/min. GC Q-TOF MS, and Auto MS-MS data were processed with Mass Hunter qualitative analysis software (v. B.06.00 SP1, Agilent Technologies Inc., USA). Agilent Mass Profiler Professional (MPP) software was used to eliminate the molecular features produce from background by deduction of data from the blank spectrum. The mass spectral deconvolution was performed by Mass Hunter Unknown Analysis Software (version B.06.00) which automatically detected peaks and deconvoluted the spectra using model ion traces.

### Statistical analysis

The results were calculated as the mean ± SD from three separate experiments (*n* = 3). The IC_50_ values of the extract and fractions for DPPH free radical scavenging and α-glucosidase inhibitory activity were determined by Graph Pad Prism 5 software. The significant differences between the means were established by ANOVA with Bonferroni post tests and Newman-Keuls multiple comparison test. Values of *P* < 0.05 were considered statistically significant.

## Results

### Yield

The average yield of methanol extract from solvent extraction by the maceration technique was found to be 12.63 ± 0.98 (as % *w*/w of *C. nutans* on a dry weight basis). The fractionation of methanolic extract produced the hexane, ethyl acetate, butanol, and residual aqueous fractions with average yields of 29.08 ± 0.070, 1.23 ± 0.03, 8.558 ± 0.0305 and 52.25 ± 1.013 (as the percentage of total methanol extract; the white precipitation from the butanol fraction was excluded from this study), respectively (Table 1)

**Table 1:** Total yield, total phenolic content and total flavonoid content of methanol extract and its different fractions

	Yield%	TPC (mg GAE/g)	TFC (mg RE/g)
Methanol	12.63 ± 0.92	7.54 ± 0.52	16.83 ± 0.85
Hexane	29.08 ± 0.70	13.31 ± 0.36	22.78 ± 0.10
Ethyl Acetate	1.23 ± 0.03	17.403 ± 0.25	13.29 ± 0.04
Butanol	8.558 ± 0.30	4.105 ± 0.75	10.04 ± 0.56
R. Aqueous	52.25 ± 1.01	1.32 ± 0.02	9.53 ± 0.088

Each value in the table is represented as mean ± SD (*n* = 3)

.

### Total phenolic content (TPC) and total flavonoid content (TFC)

The total phenolic content of the extract and all the fractions were, reported as gallic acid equivalents per g of dried sample. The TPC of the methanol extract was 7.54 ± 0.02 mg GAE/g, whereas the ethyl acetate fraction had the highest TPC (17.403 ± 0.025 mg GAE/g) among all the fractions. The methanolic extract had a TFC of 16.83 ± 0.085 mg Rutin Equivalent/g of dried materials and the hexane fraction contained the maximum TFC (22.78 ± 0.01 mg RE/g) compared with all other fractions. The TPC and TFC of methanolic extract and the different fractions are presented in Table 1.

### DPPH free radical scavenging activity

**Fig. 1:**
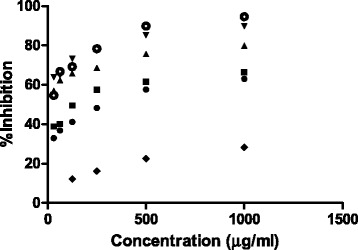
DPPH Free Radical Scavenging Activity at Different Concentration of Methanol Extract and its Fractions. The IC_50_ values of Methanol Extract and its Hexane, EoAC, BuOH, R. aqueous Fractions are 0.3302 mg/ml, 0.1527 mg/ml, 0.2691 mg/ml, 0.2517 mg/ml, 0.585 mg/ml and 0.1927 mg/ml, respectively. Legends:  MeOH,  Hexane,  EoAC,  BuOH,  R. Aqueous,  Quercitin

The DPPH free radical scavenging is a commonly used method to assess antioxidant activity in plant samples. The antioxidant activity of the samples was examined at six different concentrations (31.25, 62.5, 125, 250, 500 and 1000 μg/mL). In this study, all the samples dose- dependently scavenged DPPH radicals, except for the residual aqueous fraction. The ethyl acetate fraction showed the highest DPPH scavenging activity, at 79.98 ± 0.31% (IC50: 0.2691 mg/mL) whereas the lowest scavenging ability was observed in the methanol extract, at 63.07 ± 0.118% (IC50: 0.3302 mg/mL). The antioxidant activity of the methanol extract and its derived fractions was ranked as follows: ethyl acetate > butanol > hexane > methanol > residual aqueous fraction. The DPPH free radical scavenging activity at different concentration of methanol extract and fractions is shown in Fig. 1.

### Ferric reducing antioxidant power assay

The donation of electrons by the reduction of ferric cyanide complex in the ferrous form specifies the reducing capacity of experimental samples. Essentially, this method considers the total antioxidant capacity of a sample through the oxidation-reduction potential. The methanol extract and ethyl acetate fraction exhibited the highest FRAP values of 141.89 ± 0.874 and 133.6 ± 0.29 AAE μg/g respectively (Table 2). The reducing power of the extract and fractions was ranked as follows: methanol > ethyl acetate > butanol > hexane > residual aqueous.

**Table 2:** DPPH scavenging activity, ferric reducing antioxidant power and α-Glucosidase inhibitory activity of methanol extract and its different fractions

	DPPH%	FRAP (μg AAE/g)	AGI%
Methanol	63.07 ± 0.11^a^	141.89 ± 3.87^**/*^	58.21 ± 0.8^a^
Hexane	66.35 ± 0.09^b^	130.56 ± 2.43^*/*^	69.94 ± 0.81^b^
Ethyl Acetate	79.98 ± 0.31^c^	133.6 ± 3.29^*/*^	70.76 ± 0.49^c^
Butanol	78.47 ± 0.26^c^	132.76 ± 3.72^**/*^	72.16 ± 1.12^d^
R. Aqueous	28.32 ± 0.27^d^	12.11 ± 0.31^ns^	41.86 ± 0.37^e^

Each value in the table is represented as mean ± SD (*n* = 3). Means within each column with different letters (^a-e^) differ significantly (*p* < 0.05) according to ANOVA with Newman-keuls multiple comparison test when compared with standard. For FRAP compared total phenolic and total flavonoid content where **P < 0.01, *P < 0.05, ^ns^ *P* > 0.05 according to ANOVA with Bonferroni posttests

### α-Glucosidase inhibitory activity

**Fig. 2:**
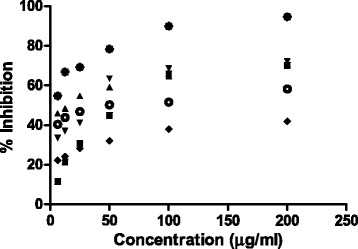
α-Glucosidase Inhibitory Activity at Different Concentration of Methanol Extract and its Fractions. The IC_50_ values of Methanol Extract and its Hexane, EoAC, BuOH, R. aqueous Fractions are 0.06139 mg/ml, 0.04457 mg/ml, 0.05369 mg/ml, 0.3747 mg/ml, 0.0635 mg/ml and 0.03854 mg/ml, Respectively. Legends:  MeOH,  Hexane,  EoAC,  BuOH,  R. Aqueous,  Quercitin

The α-glucosidase inhibitory activity of the methanol extract and the fractions was assessed at six different dilutions (6.25, 12.5, 25, 50, 100 and 200 μg/mL) (Fig. 2). The highest potential α-glucosidase inhibitory activities were found in the butanol and ethyl acetate fractions with inhibition of 72.16 ± 1.0041% (IC50: 37.47 μg/mL) and 70.76 ± 0.4974% (IC50: 53.69 μg/mL) (Table 2). The efficacy of the α-glucosidase inhibition of the extract and various fractions was ranked as follows: butanol > ethyl acetate > hexane > methanol > residual aqueous. The standard quercetin was used as positive control and showed a maximum α-glucosidase inhibitory activity of 94.63 ± 1.21% (IC50: 38.54 μg/mL).

### GC Q-TOF MS analysis

GC Q-TOF MS analysis was conducted to identify the phytoconstituents of the methanol extract and the four fractions. The spectral data were integrated with mass retention index of NIST14.L library. This study only considered those compounds with matching library scores with similarity index of 70%. The biological potential of the identified compounds was compared with previously reported results (Table 3). The major phytochemicals were found in the methanol extract and the fractions, except residual aqueous. The identified phytochemicals belonged mainly to the classes of α-hydroxyl acids, dicarboxylic acids, terpenes, phenolics, inositols, fatty acids, coumarins, glycosides, phytosterols, polysiloxanes and polyols. The phytochemical profiling of *C. nutans* revealed that experimental samples were abundant in the phenolic and terpenoids compounds.

**Table 3:** Identified metabolites in methanol extract and its different fractions

MeOH	Mass	Formula (DB)	Score (Lib)	m/z	RT
Glyceric acid ^rq^	322.145	C_12_H_30_O_4_	82.1	73.03153	22.04
Malic acid ^hex, rq^	350.14	C_13_H_30_O	82.63	73.03153	31.411
Erythritol ^hex, bu., rq^	410.216	C_16_H_42_O_4_	83.96	73.03153	33.044
Ribitol ^hex^	512.266	C_20_H_52_O_5_	90.44	73.03153	49.243
Neophytadiene	278.297	C_20_H_38_	81.5	67.0393	53.357
Mannitol ^bu^	614.316	C_24_H_62_O_6_	86.43	73.03153	56.957
Gluconic acid	628.295	C_24_H_60_O_7_	72.71	73.03153	58.972
Palmitic acid ^rq^	328.28	C_19_H_40_O_2_	75.99	117.0168	59.131
Rhamnose ^ea., bu., rq^	452.227	C_18_H_44_O_5_	71.07	73.03153	59.918
Myo-Inositol ^hex, ea., bu^	612.301	C_24_H_60_O_6_	81.27	73.03153	61.069
Oleic Acid ^hex^	354.295	C_21_H_42_O_2_	74.58	75.01034	63.292
Stearic acid ^hex, ea., bu., rq^	356.311	C_21_H_44_O_2_	75.89	117.0168	63.921
1-Monopalmitin ^hex, ea., bu., rq^	474.356	C_25_H_54_O_4_	86.25	371.2661	76.818
Hexane	Mass	Formula (DB)	Score (Lib)	m/z	RT
Glycolic acid ^ea., bu., rq^	220.095	C_8_H_20_O_3_	78.81	147.0445	8.457
Glycerol ^hex, bu^	308.166	C_12_H_32_O_3_	86.8	73.03193	18.785
Myristic acid	300.248	C_17_H_36_O_2_	77.37	117.0175	53.071
Hexadecanoic acid ^bu^	270.256	C_17_H_34_O_2_	80.98	87.02704	55.509
Cyclononasiloxane ^ea., bu^	666.169	C_18_H_54_O_9_	78.42	73.03193	64.886
Squalene	410.391	C_30_H_50_	76.25	81.05419	83.616
Sulfurous acid	376.301	C_21_H_44_O_3_	79.63	57.05663	85.537
β-Tocopherol	488.405	C_31_H_56_O_2_	78.3	488.3697	87.803
1-Tetracosanol	426.426	C_27_H_58_O	74.64	411.3688	87.824
γ-Tocopherol	488.405	C_31_H_56_O_2_	76.32	73.03193	88.004
Lupeol	426.386	C_30_H_50_O	78.1	189.1404	98.854
EoAC	Mass	Formula (DB)	Score (Lib)	m/z	RT
Lactic Acid ^rq^	234.111	C_9_H_22_O_3_	70.76	73.03193	7.867
Hexanoic acid	188.123	C_9_H_20_O_2_	82.18	73.03193	8.127
Oxalic acid	234.074	C_8_H_18_O_4_	74.24	147.0437	11.408
Benzoic acid	194.076	C_10_H_14_O_2_	89.19	135.0956	16.142
Benzeneacetic acid	208.092	C_11_H_16_O_2_	82.37	73.03193	19.06
Tyrosol	282.147	C_14_H_26_O_2_	83.45	179.063	35.281
Arabinitol ^bu., rq^	512.266	C_20_H_52_O_5_	87.01	73.03193	49.249
Vanillic acid	312.121	C_14_H_24_O_4_	81.66	297.1034	49.77
4-Coumaric acid	308.126	C_15_H_24_O_3_	81.65	293.1079	50.764
p-Coumaric alcohol	294.147	C_15_H_26_O_2_	75.26	73.03193	51.005
Azelaic acid	332.184	C_15_H_32_O_4_	85.4	73.03193	51.378
Tryptophol	305.163	C_16_H_27_NO	73.32	202.1438	54.561
Syringic acid	342.132	C_15_H_26_O_5_	76.23	327.0037	55.145
Heptasiloxane	532.184	C_16_H_48_O_6_	70.08	355.0375	87.993
D-Xylose ^bu^	438.211	C_17_H_42_O_5_	74.23	191.0664	88.537
BuOH	Mass	Formula (DB)	Score (Lib)	m/z	RT
Propylene glycol	220.131	C_9_H_24_O_2_	72.85	73.03256	13.928
Dulcitol	614.316	C_24_H_62_O_6_	73.74	73.03256	56.96
Octadecanoic acid	284.272	C_18_H_36_O_2_	71.73	87.02775	62.021
Residual Aqueous	Mass	Formula (DB)	Score (Lib)	m/z	RT
Butanedioic acid	262.106	C_10_H_22_O_4_	78.34	147.0448	20.507
Xylitol	512.266	C_20_H_52_O_5_	75.32	73.0321	49.24

Subscript with hex-hexane fraction, ea.- ethyl acetate fraction, bu.-butanol fraction, rq-residual aqueous fraction represents the identified compound in that fraction

## Discussion

In the current study, we fractionated the crude extract of *C. nutans* using polar and non-polar solvents to obtain phytoconstituent rich biologically active standardized fractions and produced a comprehensive metabolite profiling. The average yield, TPC, and TFC of different samples are presented in Table 1. In order to achieve high quality fractionation, the purification of the extract essentially be purified to remove undesirable components could, enhance the biological activity for future pharmaceutical applications. Therefore, it is crucial to select an appropriate extraction process, as affects the characterization of secondary metabolites [32]. Plant phenolics are one of the most abundant vital group of phytochemicals and are widely known for their antioxidant and radical scavenging activity with potential impacts on human health. The phenolics are natural antioxidants which can control oxidative stress-related degenerative diseases. The adverse effects of oxidative stress have been found to be controlled by the antioxidant activities of this group of bioactive compounds. Therefore, numerous experimental studies have been designed to elucidate the antioxidant and other activities of these compounds that can inhibit certain pathological and degenerative complications [33].

**Fig. 3:**
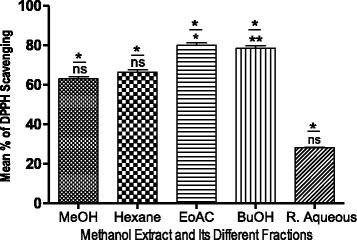
Effect of TPC and TFC on DPPH Free Radical Scavenging Activity. Bars with **P < 0.01, *P < 0.05 are Significantly Correlated whereas ^ns^ *P* > 0.05 Denotes Non significant Correlation According to Bonferroni Posttests (The Numerator on Bar Represent the Correlation with TPC and Denominator with TFC)

As presented in Fig. 1, dose-dependent DPPH radical scavenging capacity was examined in the methanol extract and fractions, in which the standard compound (quercetin) produced the maximum antioxidant activity (IC50: 0.1927 mg/mL). The methanol extract, hexane and residual aqueous fractions were statistically significant when compared with quercetin (Table 2). This research work also revealed a strong agreement between the antioxidant activity of samples when measured by the TPC and TFC. Among all the fractions, the ethyl acetate fraction had the highest DPPH free radical scavenging activity and was rich in phenolic and flavonoid content (*P* < 0.05) (Table 2). The TPC, TFC of butanol, and TPC of the hexane fraction also shown a significant correlation with antioxidant activity (*P* < 0.05) (Fig. 3). Hence, these findings support the antioxidant function of phenolics and are also in good agreement with previous research [34].

To determine the reducing power, the ferric reducing antioxidant power (FRAP), was studied for the methanol extract and the fractions of *C. nutans* to determine the reducing power; this provides a substantial indicator of antioxidant capacity. The presence of reducing agents in the samples is associated with the exertion of antioxidant activity by the cleavage of free radical chains through the donation of hydrogen atom. The statistically significant TPC and TFC values (*P* < 0.05), showed a positive correlation with the FRAP of methanol extract and fractions, except for the residual aqueous (Table 2). The methanol extract showed the highest reducing power, with a value of 141.89 ± 0.87 μg AAE/g followed by the ethyl acetate (133.6 ± 0.29), butanol (132.76 ± 0.72), hexane (130.56 ± 0.43) fractions.

An effective approach for the management of carbohydrate metabolic disorders, including diabetes mellitus type II is the inhibition of the α-glucosidase enzyme [35]. A delay in the digestion of carbohydrate plays an important role in the control of postprandial hyperglycemia, hyper insulinemia and decreases the risk of cardiovascular disease [36]. The α-glucosidase inhibitory activity of the methanol extract and fractions were significantly to the standard compound (Table 2).

Natural bioactive compounds regulate blood glucose level through obstructing the degradation of polysaccharides [37]. Plant phenolics are natural α-glucosidase inhibitors because they inhibit intestinal carbohydrate digesting enzymes owing to their protein-binding capability [38]. Recent researchers have reported the α-glucosidase inhibitory activity of numerous flavonoid compounds [39]. As shown in Fig. 2, the α-glucosidase inhibitory activity of methanol extract and the obtained fractions were evaluated at different concentrations: the strongest inhibitory concentration (IC50 37.47 μg/mL) was found in the butanol fraction compared with the standard compound. A multiple range test, between the extract, fractions and positive control is shown in Table 2; the extract and fractions were significantly different from the control (*P* < 0.05).

**Fig. 4:**
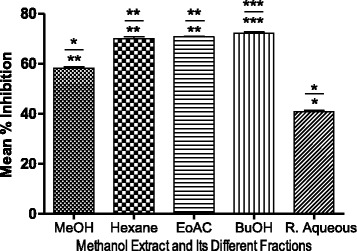
Effect of TPC and TFC on α-Glucosidase Inhibitory Activity. Bars with ***P < 0.01, **P < 0.01, *P < 0.05 are Significantly Correlated whereas ^ns^ *P* > 0.05 Denotes Non significant Correlation According to Bonferroni Posttests (The Numerator on Bar Represent the Correlation with TPC and Denominator with TFC)

The current study also revealed a significant (*P* < 0.05) correlation between the TPC and TFC with an α-glucosidase inhibitory activity of the samples (Fig. 4). The higher concentration phenolics, flavonoids and higher antioxidant activity, resulted in a higher enzyme inhibitory capacity (Table 2). The results from the current study are in good agreement with other research works, which have reported the ability of phenolic rich extracts that exhibited a high antioxidant and α-glucosidase inhibitory effect [40]. Furthermore, these results revealed a direct correlation between antioxidant activity and α-glucosidase inhibitory activity. Butanol, ethyl acetate and hexane fractions revealed similar α-glucosidase inhibitory activities of 72.16 ± 1.0041%, 70.76 ± 0.4974% and 69.94 ± 0.8115% whereas the methanol extract showed moderate inhibitory activity 58.21 ± 0.08%. The butanol fraction had significantly higher (*P* < 0.05) α-glucosidase inhibitory activity, with an IC50 value of 37.47 μg/mL, which is close to the standard quercetin compound (IC50: 38.54 μg/mL). The higher phytochemical content and antioxidant activity significantly influenced the α-glucosidase inhibitory activity of the samples (*P* < 0.05).

The most abundant identified phytoconstituents were neophytadiene, squalene, lupeol, tocopherols, vanillic acid, syringic acid, myo-inositol, glycolic acid, butanedioic acid, 4-coumaric acid, and stigmasterol. Extensive literature searches documented that the identified compounds were responsible for various biological activities, including antioxidants, antidiabetic, and anti-inflammatory chemotherapeutic agents [41]. The structural orientation of the polyphenolic compounds owing to the lactones/quinones or 4-oxo-pyrane moiety responsible for the digestive enzyme inhibitory activity [42]. It was also reported that phenolic compounds help to reduce the intestinal digestive enzymes and were able to oxidize the body fat owing to their thermogenic properties [43], whereas the terpenes and terpenoids class of compounds can act as anti-hyperglycemic agents [44].

## Conclusion

The methanol extract of *C. nutans* and the obtained solvent fractions exhibited a significant range of biological activities. The ethyl acetate and butanol fractions of the methanol extract had the highest antioxidant and α-glucosidase inhibitory activity which showed a significant correlation with the total phenolic and total flavonoid contents of the fractions. In addition, there was a strong correlation of the antioxidant activity with the α-glucosidase inhibitory potency of different samples. GC Q-TOF MS analysis showed that the presence of major phytoconstituents might be responsible for the potential antioxidant and α-glucosidase inhibitory activities of *C. nutans* extract and fractions. However, further toxicological study of the antioxidant rich fractions is necessary to verify their suitability for future pharmaceutical applications.

## Abbreviations

CN: *Clinacanthus nutans*
GC Q-TOF-MS: Gas Chromatography Quadrupole Time of Flight Mass Spectroscopy
DPPH: 2, 2-dyphenyl-1-picrylhydrazyl
TPC: Total Phenolic Content
TFC: Total Flavonoid Content
DMSO: Dimethyl sulfoxide
FRAP: Ferric Reducing Antioxidant Power
TPTZ: 2,4,6-Tripyridyl-s-Triazine
AAE: Ascorbic Acid Equivalent
ANOVA: Analysis of Variance
